# Sense of coherence moderates the relationship between social capital and oral health‑related quality of life in schoolchildren: a 10-year cohort study

**DOI:** 10.1186/s12955-022-01965-3

**Published:** 2022-04-02

**Authors:** Jessica Klöckner Knorst, Mario Vianna Vettore, Bruna Brondani, Bruno Emmanuelli, Fernanda Tomazoni, Thiago Machado Ardenghi

**Affiliations:** 1 Department of Stomatology, School of Dentistry, Universidade Federal de Santa Maria, Santa Maria, RS Brazil; 2 Department of Health and Nursing Science, University of Agder, Campus Kristiansand, Universitetsveien 25, 4630 Kristiansand, Norway; 3 Department of Pediatric Dentistry and Orthodontics, School of Dentistry, Universidade de São Paulo, São Paulo, SP Brazil

**Keywords:** Brazil, Children, Moderating effect, Oral Health, Quality of life, Sense of coherence, Social capital

## Abstract

**Background:**

This study aimed to evaluate the moderating effect of sense of coherence (SOC) on the relationship between social capital and oral health-related quality of life (OHRQoL) among schoolchildren.

**Methods:**

A cohort study was conducted in the city of Santa Maria, Brazil, involving children aged 1–5 years at baseline who were reassessed after 10 years in adolescence (11–15 years-old). Social capital was assessed at baseline and follow-up through social networks and social trust. Sense of coherence scale (SOC-13) and the short form of the Child Perceptions Questionnaire 11–14 (CPQ11–14) were measured at 10-years follow-up. Demographic and socioeconomic characteristics, and dental caries were also evaluated. Moderating effect of SOC on the relationship between social capital and OHRQoL was tested using multilevel adjusted Poisson regression analysis and simple slope test.

**Results:**

From the 639 subjects assessed at baseline, 429 were reassessed at follow-up (cohort retention rate 67.1%). Moderate and high levels of SOC demonstrated a moderating effect on the relationship between social capital and OHRQoL. Among individuals who presented low social capital at baseline and follow-up, those who had high SOC reported, respectively, an impact 63% and 70% lower on OHRQoL when compared to those with low SOC. The greatest margin effect was observed in individuals with low social capital and low SOC at follow-up (24.25; *p* < 0.05).

**Conclusion:**

Our findings suggest that SOC moderates the negative impact of low social capital on poor OHRQoL in schoolchildren.

## Introduction

Oral health inequalities remain a worldwide public health problem [1]. Currently, the focus is beyond the clinical measures of the dental diseases, and the patient's self-perception has been considered, especially on how oral health conditions affect their well-being and quality of life [2]. In this context, oral health-related quality of life (OHRQoL) measures have been widely advocated as an adjunct to clinical parameters in public policy planning and in the assessment of oral health strategies [3]. Clinical and socioeconomic conditions have been associated with OHRQoL [4, 5]. In addition, contemporary approaches recognize the importance of the salutogenic model [6] and social capital [7] on planning oral health promotion actions and strategies to enhance OHRQoL.

Social capital has been described as the characteristics of the social structure, such as levels of trust and reciprocity, or individual social networks that act as resources accessed by individuals that can facilitate collective action [8, 9]. High social capital may act as a protective factor on oral health buffering the effects of stress through the perception of mutual social support, as well as through feelings of security and belonging [10]. Other individual resources, such as sense of coherence (SOC), can also interact with a person’s coping style and social capital [11, 12]. Moreover, psychosocial mechanisms, including SOC, are central elements in the theoretical pathways developed to explain the relationship between social capital and oral health [10, 11].

SOC is defined as a global orientation that allows people to manage stress, identify their internal and external environments and find solutions for their health [6]. In this sense, SOC variations may explain why some individuals remain healthy even after experiencing stressful circumstances in life [6]. SOC has been associated with different aspects of health and disease. For instance, individuals with high SOC had less dental caries and dental pain [13], as well as better self-perceived health and better OHRQoL [14]. Previous studies have shown a moderating effect of SOC on the association between general quality of life and OHRQoL even when clinical conditions and symptoms were considered [14, 15]. In this context, SOC might be an important psychosocial factor that can act as a moderator on the relationship between social capital and oral health outcomes.

The relationship between social life the characteristics, SOC and OHRQoL in children and adolescents has been demonstrated [5, 11, 16]. However, to our best knowledge, the association between social capital and OHRQoL considering the moderating effect of SOC in these age groups has not yet been explored. Children is a relevant population to investigate the above-mentioned links since SOC is under development until early adulthood [6]. In addition, SOC can be an important ally to promote effective strategies to improve oral health. Thus, this study aimed to evaluate whether SOC modifies the association between social capital and OHRQoL from childhood to adolescence. We hypothesize that adolescent’s OHRQoL are influence by low levels of social capital during childhood and adolescence according to different levels of SOC. It was anticipated that individuals with low social capital and high SOC would have better OHRQoL than those with low social capital and low SOC.

## Methods

### Ethical aspects

This project was approved by the Research Ethics Committee (CEP) of the Federal University of Santa Maria (protocol CAAE 11765419.1.0000.5346). All participants agreed to participate in the study and their parents signed an informed consent form in both phases of the study.

### Study design and participants

This is a cohort study with 10 years of follow-up. Baseline (T1) collection was carried out in 2010 during the National Children's Vaccination Day in Santa Maria, Brazil. The estimated population of the city in 2010 was 263,403 inhabitants, which included 27,520 children up to 5 years old. The recruitment of the sample occurred in all 15 healthcare centres that had a dental office distributed in different neighbourhoods of the city. A systematic approach was adopted to select the children in the vaccination row. About 639 children up to 5 years old were evaluated. Additional information about the sample selection process is available elsewhere [17].

The cohort was followed and participants were reassessed in 2012 (2 years), 2017 (7 years), and 2020 (10 years). This study used data from baseline (T1) and 10-year follow up (T2). Data collection of the latter period was conducted between October 2019 and January 2021. Due to the COVID-19 pandemic, data collection at T2 was interrupted from March to September 2020 [18]. At T2, adolescents were searched at schools where they were enrolled, through telephone calls, and, if necessary, through online social networks such as WhatsApp and Facebook.

A post hoc power calculation was performed considering the final sample size and the estimates obtained from our sample. A sample of 429 participants, alpha error probability of 0.05, mean score of CPQ11-14 of 7.6 (SD = 6.3) for the non-exposed group (high SOC), and mean score of 19.5 (SD = 10.3) for the exposed group (low SOC), resulted in a sample power of 100%.

### Data collection and variables

Data was collected at the dental office of the healthcare centres at T1 and at the participant’s homes or schools at T2 through clinical examinations and interviews using a structured questionnaire, following the international protocol for health surveys [19–22].

Social capital was evaluated at T1 and T2. In the former period, social capital was measured considering parents/legal guardians social networks using the following questions: (a) “How often have you visited friends and neighbours in the last 12 months?”; (b) “How often have you visited family members in the last 12 months?”; and (c) How often do you participate in group religious activities, with the following response options: (0) at least once a month; (1) less than once a month or never. Social capital was evaluated through adolescent social networks and social trust at T2, using the following questions: (a) “How often do you participate in group religious activities” (0) at least once a month or (1) less than once a month or never; (b) “Do you participate in any group volunteer work?” (0) yes or (1) no and c) "Do you think your friends and neighbours are trustworthy?” (0) yes or (1) no. The items used to assess social capital at T1 and T2 are considered reliable proxies of social capital according to the literature [5, 23]. Participants were classified as with high social capital (at least one source of social network or trust) or low social capital (absence of any source of network or trust) for analytical purposes as previously suggested [24, 25].

OHRQoL was assessed at T2 using the short version of the Child Perceptions Questionnaire 11–14 (CPQ11-14). The questionnaire was previously adapted and culturally transcribed for 5-years-old Brazilian children [20]. The short version of CPQ11-14 is composed of 16 questions, grouped into 4 domains: oral symptoms, functional limitation, social well-being, and emotional well-being. Each item is followed by a five-point Likert score: (0) “never”; (1) “once or twice”, (2) “sometimes”, (3) “often”; and (4) “every day/almost every day”. OHRQoL scores are computed by summing code responses with a final score ranging from 0 to 64. The higher the score, the worse the OHRQoL.

Adolescent SOC was assessed according to the Brazilian short version of the SOC-13 scale, originally developed by Antonovsky (1987) [6, 21]. The questions are divided into three components: comprehensibility, manageability and meaningfulness. The response options follow a 5-point Likert scale varying from 1 to 5. The first two items of the SOC-13 scale include the following prompts: (1) “What you do daily is…”; and (2) “Until today your life has been…” The response options for the first item vary from (1) “an enormous suffering and annoyance” to (5) “a great pleasure and satisfaction”, and from (1) “with no aim” to (5) “full of aims” for the second item. The following items relate to coping in everyday life and the response options range from (1) “never” to (5) “always”. The last item refers to the perception of the importance given to life events, and the answers can vary from (1) “totally wrong” to (5) “totally right”. The item codes are added to obtain the final score, which can vary from 13 to 65. Higher scores indicate stronger levels of SOC. For data analysis, SOC was categorized using the mean (36.2) and + -1SD (8.0) of the sample, according to previous literature [15]. Thus, the participants were categorized as of low (up to score 28.2), moderate (28.3 to 36.1), and high SOC (from 36.2 onwards).

Sociodemographic characteristics and dental caries were measured at baseline and follow-up as possible confounders. Demographic characteristics included sex (girls or boys) and age (in complete years). Skin colour was self-reported through the question: “What is your / your child race or skin colour?” at T1 (to parents) and T2 (to child), whit the response options: (1) “white”, (2) “brown”, (3) “black”, (4) “yellow” or (5) “indigenous”, according to the criteria proposed by the Brazilian Institute of Geography and Statistics (IBGE) [26]. For data analysis, skin colour was dichotomized as whites (0) or on-whites (2,3,4,5). Maternal education was assessed according to years of schooling completed with approval and dichotomized into < 8 years (up to primary school) or ≥ 8 years (incomplete secondary school or more). Monthly family income in the previous month was collected and categorized as > 1 Brazilian minimum wage (BMW) or ≤ 1 BMW. One BMW corresponded to 200 USD when the data was collected. Dental caries was assessed by the diagnostic criteria of the International Caries Detection and Assessment System (ICDAS) [22]. The number of teeth with untreated dental caries (ICDAS code 3, 5, or 6) was considered in the analysis. Surfaces with ICDAS stages 0, 1, 2 and 4 were classified as caries free. The dental exams were performed under artificial light, using a plain dental mirror, periodontal probe (CPI; “ballpoint”) and gauze. The examiners were previously trained and calibrated (Kappa > 0.70).

### Statistical analysis

Data analysis was performed using STATA 14.0 statistical software (StataCorp. 2014. Stata Statistical Software: Release 14.0. College Station, TX: StataCorp L). The comparisons between participants who were assessed at follow up and dropouts, and between individuals assessed before and during the COVID-19 pandemic were evaluated by the chi-square test (qualitative variables) and the t-test (quantitative variables).

**Fig. 1:**
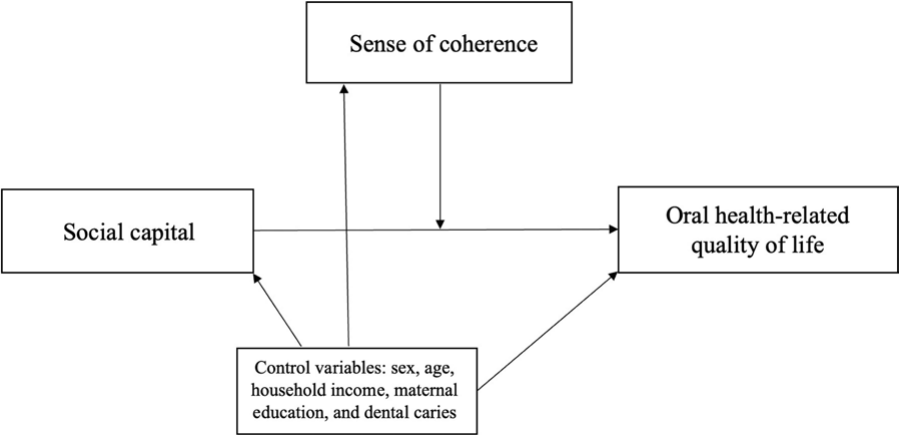
The logic map of the moderation effects

The study outcome was OHRQoL measured through the CPQ11-14 total scores. Unadjusted and adjusted Multilevel Poisson regression analysis was performed to evaluate the moderating effect of SOC on the relationship between social capital and OHRQoL. Moderation effects occur when the relationship between two variables vary according to a third variable, which is referred as the moderator variable. The effect of a moderating variable is statistically characterized as interaction; that is, a variable that affects the direction and/or strength of the association between the dependent and independent variables [27]. Our data was tested in multiplicative interactions scale to verify the modification of the effect, as in previous studies [14, 15]. The logic map of the moderation effects is presented in Fig. 1. The interaction in different categories was considered as follows: 0 = low social capital × low SOC; 1 = high social capital × low SOC; 2 = high social capital × high SOC; and 3 = low social capital × high SOC). Sociodemographic characteristics and dental caries variables with *p* ≤ 0.20 in the unadjusted analysis were included in the adjusted model as possible confounders. The multilevel structure of analysis considered individuals (level 1) nested into 15 neighbourhoods (level 2). Multilevel analysis considered the individual sampling weights when adjusting for survey design. The individual sampling weights consider the inverse of the probability of selecting the adolescent, once the adolescent’s neighbourhood has already been selected. All the analysis were conducted using the *svy* command for fitting multilevel models to survey data in Stata. The results are presented in Rate Ratio (RR) and 95% confidence intervals (95% CI).

The simple slope test was conducted afterwards once the hypothesized moderation effects were statistically significant to obtain the simple margins of predicted values by each level of the categorical moderator. This procedure allows the calculation of the conditional effect of social capital on OHRQoL according to SOC levels (moderator), generating a confidence interval and *p* values [27]. A significance level of 0.05 was considered.

## Results

Of the 639 subjects assessed at baseline, 429 were reassessed at 10-years follow-up (cohort retention rate = 67.1%). The reasons for losses to follow-up were impossibility of finding the adolescent (n = 184); move to another city (n = 19); or refusal to participate (n = 7). There were no significant differences of characteristics between individuals who completed the 10-years follow up and dropouts, nor between those evaluated before and during the COVID-19 pandemic (*p* > 0.05).

Table 1 reports the descriptive characteristics of the sample. The sample was balanced between boys and girls, and most individuals reported white skin colour in both assessments. The mean age was 2.8 (SE 0.1) and 12.5 (SE 0.1) years old at baseline and follow-up. Most individuals presented monthly family income higher than 1 BMW (70.8%) and mothers with more than 8 years of schooling at T1. The majority of participants presented high levels of social capital at T1 and T2, and about 7.9% of the sample presented low SOC at T2. The mean of CPQ11-14 total scores at follow-up was 11.2 (SE = 0.6).

**Table 1:** Demographic, socioeconomic, psychosocial, and oral health variables of the sample at baseline and follow-up

Variables	Baseline (T1) 2010 (n = 639)	Follow-up (T2) 2020 (n = 429)	*p* value
*Demographic and socioeconomic variables*			
Sex [n (%)]			0.227
Boys	322 (49.0)	209 (49.8)	
Girls	317 (51.0)	220 (50.2)	
Age [mean (SE)]	2.8 (0.1)	12.5 (0.1)	0.101
Skin color			0.158
White	501 (80.5)	215 (48.5)	
No-white	137 (19.5)	211 (51.5)	
Household income in BMW [n (%)]			0.109
≤ 1BMW	129 (19.0)	110 (29.2)	
> 1BMW	473 (81.0)	264 (70.8)	
Maternal education [n (%)]			0.669
≥ 8 years	357 (54.3)	285 (69.6)	
< 8 years	275 (45.7)	110 (30.4)	
*Psychosocial variables*			
Social capital [n (%)]			0.472
High	479 (75.6)	315 (73.5)	
Low	154 (24.4)	110 (26.5)	
Sense of coherence [n (%)]			–
Low	–	35 (7.9)	
Middle		145 (32.6)	
High		249 (59.5)	
*Oral health variables*			
Untreated dental caries [n (%)]			0.773
Absent	408 (61.6)	300 (69.4)	
Present	231 (38.4)	128 (30.6)	
CPQ11-14 [mean (SE)]	–	11.2 (0.6)	–

BMW, Brazilian minimum wage; SE, standard deviation; CPQ, child perception questionnaire

^*^Taking into account the sampling weight; Values lower than 639 or 429 are due to missing data

^†^Comparison between followed and dropouts’ individuals

Table 2 shows the multilevel unadjusted analysis considering the interaction of social capital at T1 and T2 and SOC at T2 on CPQ11-14 total scores at follow-up. In the crude analysis, individuals with low social capital at T2 (1.16; 95% CI 1.01–1.32) had greater likelihood of poor OHRQoL. Individuals with high SOC were more likely to report better OHRQoL (RR 0.39; 95% CI 0.36–0.46). The majority of categories of the interaction term social capital × SOC were associated with OHRQoL considering low social capital and low SOC as the reference category.

**Table 2:** Unadjusted association of social capital at T1 and T2 and sense of coherence with overall CPQ11-14 scores at follow-up

Variables	OHRQoL (CPQ11-14)	
	RR (95% CI)*	*p* value
Social capital (T1)		
High	1 (reference)	
Low	1.12 (0.79–1.58)	0.507
Social capital (T2)		
High	1 (reference)	< 0.001
Low	1.16 (1.01–1.32)	
Sense of coherence (T2)		
Low	1 (reference)	
Middle	0.73 (0.64–0.83)	< 0.001
High	0.40 (0.35–0.45)	< 0.001
Interaction variables		
Social Capital (T1) × Sense of coherence (T2)		
Low × Low	1 (reference)	
High × Low	0.99 (0.78–1.27)	0.993
High × Middle	0.71 (0.55–0.91)	< 0.001
High × High	0.40 (0.33–0.51)	< 0.001
Low × Middle	0.81 (0.55–1.17)	0.274
Low × High	0.37 (0.29–0.47)	< 0.001
Social Capital (T2) × Sense of coherence (T2)		
Low × Low	1 (reference)	
High × Low	0.69 (0.42–1.13)	0.146
High × Middle	0.57 (0.35–0.91)	< 0.005
High × High	0.31 (0.22–0.43)	< 0.001
Low × Middle	0.58 (0.44–0.76)	< 0.001
Low × High	0.32 (0.20–0.51)	< 0.001

^*^Taking into account the sampling weight; OHRQoL, oral health-related quality of life; RR, rate ratio; CI, confidence interval; T1, baseline; T2, 10-years follow-up;

The results of the moderation analysis after adjustment for confounders are presented in Table 3. In general, moderate and high levels of SOC demonstrated a moderating effect on the relationship between social capital and OHRQoL. Among individuals who presented low social capital at T1 and T2, those who had higher SOC reported, respectively, an impact 63% and 70% lower on OHRQoL than those with low SOC. Considering those with high social capital at T1 and T2, those who had higher SOC reported an impact 55% and 71% lower on OHRQoL than those with low SOC. Regardless of the SOC level, the relationship between SOC and OHRQoL was also lower among individuals with high social capital.

**Table 3:** Adjusted analysis of the interaction of social capital at T1 and T2 and sense of coherence on overall CPQ11-14 scores at follow-up

Interaction variables	OHRQoL (CPQ11-14)	
	RR (95% CI)*	*p* value
Social Capital (T1) × Sense of coherence (T2)		
Low × Low	1 (reference)	0.447
High × Low	1.11 (0.84–1.48)	0.154
High × Middle	0.81 (0.62–1.07)	< 0.001
High × High	0.45 (0.35–0.57)	0.486
Low × Middle	0.87 (0.61–1.26)	< 0.001
Low × High	0.37 (0.30–0.44)	
Social Capital (T2) × Sense of coherence (T2)		
Low × Low	1 (reference)	
High × Low	0.64 (0.43–0.93)	< 0.005
High × Middle	0.55 (0.39–0.77)	< 0.001
High × High	0.29 (0.23–0.35)	< 0.001
Low × Middle	0.58 (0.48–0.69)	< 0.001
Low × High	0.30 (0.20–0.44)	< 0.001

^*^Taking into account the sampling weight and adjusted by sex, skin color, age, household income, and untreated dental caries; OHRQoL, oral health-related quality of life; RR, rate ratio; CI, confidence interval; T1, baseline; T2, 10-years follow-up

**Fig. 2:**
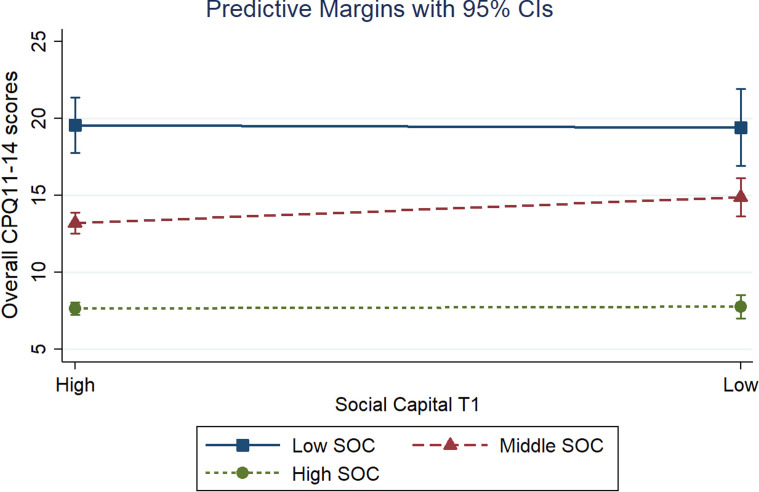
Predictive marginal effects between social capital at baseline and overall CPQ11-14 scores according to different levels of sense of coherence

**Fig. 3:**
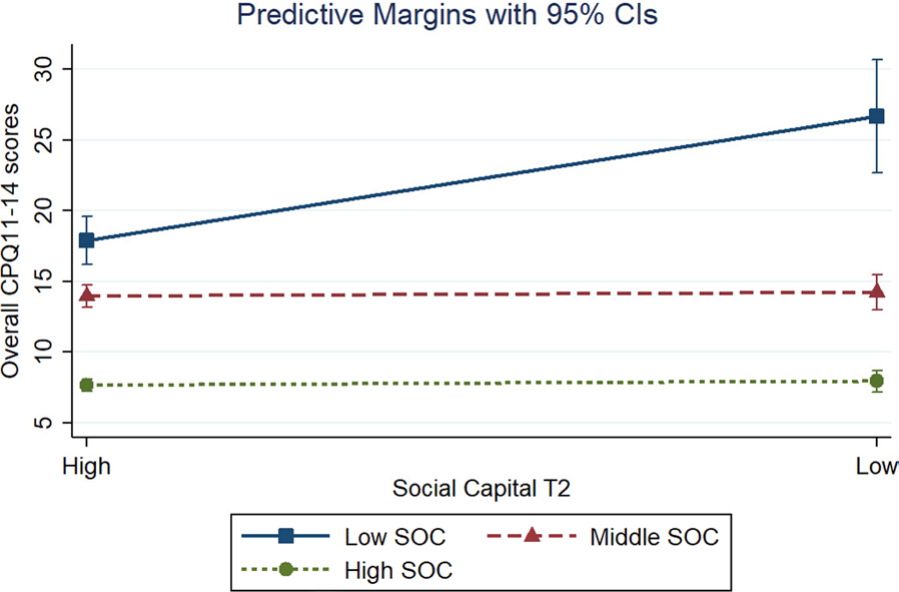
Predictive marginal effects between social capital at follow-up and overall CPQ11-14 scores according to different levels of sense of coherence

Figures 2 and 3 presents the predictive marginal effects between social capital at T1 and T2 and CPQ11-14 total scores according to different levels of SOC. According to the figures, the differences in predictive margins are visible only considering high levels of social capital at follow-up (cross-sectional interaction). However, the simple slope test (Table 4) indicated that the negative effects of low social capital on OHRQoL were statistically significant across different levels of SOC levels (low, moderate, and high) in both assessments (T1 and T2). The greatest margin effect was observed in individuals with low social capital and low SOC at T2 (24.25; *p* < 0.05).

**Table 4:** Predictive marginal effects between the social capital and OHRQoL according to different levels of sense of coherence among individuals with low social capital

Interaction variables	OHRQoL	
	Margin (95% CI)	*p* value
Low social capital at T1		
Low SOC	19.41 (16.92–21.90)	< 0.001
Middle SOC	14.89 (13.64–16.13)	< 0.001
High SOC	7.76 (6.99–8.52)	< 0.001
Low social capital at T2		
Low SOC	24.25 (20.8–27.6)	< 0.001
Middle SOC	13.61 (12.5–14.8)	< 0.001
High SOC	7.93 (7.21–8.65)	< 0.001

SOC, sense of coherence; CI, confidence interval; T1, baseline; T2, 10-years follow-up

## Discussion

This study aimed to evaluate the moderating effect of SOC on the association between social capital and OHRQoL. Our findings confirm the conceptual hypothesis that high SOC could attenuate the impact of low social capital on poor OHRQoL. In addition, the greatest moderation effect of SOC was observed in the interaction with social capital at follow-up. Despite the relationship between the characteristics of social life, SOC and OHRQoL in children and adolescents has already been researched [5, 11, 15], such association considering the moderating effect of SOC has not been explored yet.

Considering the predictor variables separately, individuals with low social capital at baseline and at follow-up presented poorer OHRQoL at follow-up. This finding corroborates previous cross-sectional and cohort studies [5, 15, 28]. In our study, social capital was measured through proxies such as social networks and perception of trust. Thus, the present findings can be explained since individuals with more social networks and trust are subject to peer and more likely to adopt healthy behaviours [10, 29] that may influence OHRQoL. Moreover, these individuals are more likely to use dental services [10, 29], which in turn can be related to better OHRQoL. Greater social capital can also benefit health acting as a protective factor buffering the effects of stress through feelings of security, belonging, and social support, and consequently impacting on self-perceived health and quality of life [10, 29]. Thus, individuals with low levels of social capital tend to report more oral impacts on their quality of life.

Among individuals with low social capital, those with moderate and high levels of SOC showed lower odds of worse OHRQoL than those with low SOC. Previous studies have shown a moderating effect of SOC on general and OHRQoL, considering other predictors, such as the need for dental prostheses and dental caries [14, 21]. Furthermore, it has been suggested that SOC interacts with a person's natural coping style and social support [11, 12]. Subjects with high SOC assess situations in a more comprehensive way, see life events and health-disease problems as challenges worthy of effort, perceive available resources more easily, and use them to cope with stress when necessary [6]. Previous studies have reported that high SOC was associated with better normative and subjective oral health outcomes [13, 14, 30]. Thus, despite having low social capital, more resilient individuals tend to feel less affected by oral problems, and consequently, present a better OHRQoL.

Our findings also indicated that the negative effects of low social capital on OHRQoL were statistically significant across different levels of SOC in both assessments (baseline and follow-up). However, the greatest moderation effect of SOC was observed in the interaction with social capital at follow-up (cross-sectional interaction). Possible explanations for this finding include the assessment of social capital by parents at baseline and by the adolescents at follow-up. In addition, social capital may have changed over time. Parent’s social capital may be different from the adolescent's social capital, since social capital may vary according to time, gender, and personal experiences [31]. In addition, since SOC interacts with a person's natural coping style and social capital, the extent to which these elements are available is one of the main determinants related to the development of SOC [11]. In this sense, the protective effect provided by the interaction between the social capital reported by adolescents and their SOC on OHRQoL may be more strongly linked in the same stage of life, which is in agreement with our results.

Our findings must be interpreted with caution due to some limitations. First, we assessed social capital through indicators or proxies, which may not give a complete measure of the construct. However, these indicators have been commonly used in previous studies [5, 22]. In addition, possible changes of SOC over the study period was not assessed since the construct was only investigated at follow-up. Finally, follow-up data collection was affected by the COVID-19 pandemic, which may have led to information bias among individuals who were assessed before and during this period. However, sensitivity analysis demonstrated that this issue did not affect our findings. Moreover, postponing data collection for a post-pandemic period would possibly result in the modification of the predictors considered in this study that affect the OHRQoL. In addition, the observational nature of our study design imposes restrictions whether the observed differences are clinically relevant. Nevertheless, our findings showed that all differences in the predictive margins between social capital and overall OHRQoL scores according to different levels of SOC were greater than the minimally important difference (MID) reported in previous clinical studies that used CPQ11-14 [32, 33].

Our study also has strengths. This 10-year cohort study had a high retention rate (67.1%) and was conducted during an important period of life characterized by biopsychosocial development. Studying psychosocial factors from childhood to adolescence is extremely important, as experiences during this stage can be perpetuated throughout life [34, 35]. In addition, our study considered important psychosocial conditions, which have been explored in the previous literature [12–15, 36, 37]. In this context and based on our findings, further research is needed to evaluate interventions aiming to promote SOC and social capital, and their impact on the subjective health and oral health promotion in different population groups. Furthermore, future research aiming to examine the possible role of other modifying factors of oral health conditions, as well as high-quality trials, especially assessing the sustainability of the intervention effect, is necessary.

## Conclusion

Our findings showed that SOC may have a moderating effect on the relationship between social capital and OHRQoL. Schoolchildren with low social capital and high SOC were protected from having worse OHRQoL compared to individuals with low SOC. This finding is useful to encourage public health policies aiming to stimulate and increase SOC among individuals, especially from childhood to adolescence, once it can mitigate the harmful effects of low social capital on OHRQoL.

## Abbreviations

CPQ: Child Perception Questionnaire
OHRQoL: Oral Health-related Quality of Life
BMW: Brazilian minimum wage
WHO: World Health Organization
DMFT: Decayed, missing and filling teeth
SD: Standard deviation
SE: Standard error
SOC: Sense of coherence
RR: Rate Ratio
CI: Confidence interval
